# A 14-Year-Old Male Patient With Bone Marrow Failure Syndrome, Without Deafness, Caused by a Novel SRP72 Mutation Inherited From His Father: A Case Report

**DOI:** 10.7759/cureus.89574

**Published:** 2025-08-07

**Authors:** Dan Sun, Quanfang Luo, Chunlin Wu, Hui Deng, Miao Li

**Affiliations:** 1 Clinical Genomics Center, Wuhan Kingmed Medical Laboratory Co. Ltd., Wuhan, CHN; 2 Hematology, Nanxishan Hospital of Guangxi Zhuang Autonomous Region, Guilin, CHN; 3 Management, Wuhan Kingmed Center for Clinical Laboratory Co. Ltd., Wuhan, CHN; 4 Management, Guangxi Kingmed Center for Clinical Laboratory Co. Ltd., Nanning, CHN

**Keywords:** inherited bone marrow failure, loss of function, novel mutation, srp72 gene, teenager

## Abstract

Inherited bone marrow failure syndrome 1 (IBMFS1) is a rare autosomal dominant disorder associated with mutations in the SRP72 gene. However, mutations in this gene are exceedingly rare, and the clinical manifestations are often nonspecific, leading to delayed or misdiagnosed cases. The incidence, lifetime risk, and clinical management guidelines for SRP72-related IBMFS1 are poorly understood due to its rarity. Molecular diagnosis is essential for accurate diagnosis, treatment, and prognosis. Here, we report a novel frameshift mutation in the SRP72 gene identified in a 14-year-old patient presenting with pancytopenia. Sanger sequencing revealed that the mutation was inherited from the patient’s asymptomatic father. In vitro functional studies indicated a loss-of-function mechanism. Our case expands the mutation spectrum of the SRP72 gene and highlights the importance of genetic testing in identifying hematologic disorders.

## Introduction

Bone marrow failure syndromes (BMFS) are a group of rare, often inherited disorders characterized by the inability of the bone marrow to produce sufficient blood cells, typically manifesting as anemia, neutropenia, or thrombocytopenia [1]. These syndromes can be classified into inherited and acquired forms. Traditional diagnostic methods are often limited due to the low incidence rate and diverse clinical manifestations of inherited bone marrow failure syndromes (IBMFS). However, with the advent of next-generation sequencing (NGS), genetic testing has become increasingly important for clinical diagnosis and management, deepening our understanding of these diseases. Common IBMFS include Fanconi Anemia (FA), Dyskeratosis Congenita (DC), Shwachman-Diamond Syndrome (SDS), Diamond-Blackfan Anemia (DBA), Congenital Amegakaryocytic Thrombocytopenia (CAMT), and Severe Congenital Neutropenia (SCN) [2-5].

The *SRP72* gene encodes a crucial protein subunit of the signal recognition particle (SRP), a ribonucleoprotein complex essential for targeting secretory proteins to the endoplasmic reticulum (ER). The SRP72 protein is a key component of the SRP complex, which facilitates the co-translational targeting of nascent polypeptides to the ER [6,7]. Variants in the *SRP72* gene have been implicated in familial bone marrow failure, highlighting its importance in maintaining normal hematopoietic function.

But there were only two families with different types of variants of the *SRP72 *gene. In the first family, the mother exhibited myelodysplastic syndrome (MDS). The index case presented with aplastic anemia (AA) at the age of 14. Her two siblings had pancytopenia, and only one of them did not show hearing impairment. In the second family, the mother and daughter had a later onset of disease. The daughter was found to have thrombocytopenia and megaloblastic anemia starting at the age of 33, but her routine blood tests showed no significant abnormalities at the age of 52. Her mother was diagnosed with MDS and presented with reduced hemoglobin levels at the age of 76. The loss-of-function variants are rare in the general population, which may be overlooked in practical clinical applications.

BMFS are clinically significant due to their severe impact on hematopoiesis, leading to life-threatening complications such as infections, bleeding, and malignancies. Early diagnosis and management are crucial for improving outcomes and quality of life for affected individuals. Here, we report a novel variant in the *SRP72* gene identified in a 14-year-old patient presenting with pancytopenia. This variant was inherited from the patient's asymptomatic father. We further explore the functional implications of this mutation.

## Case presentation

**Figure 1:**
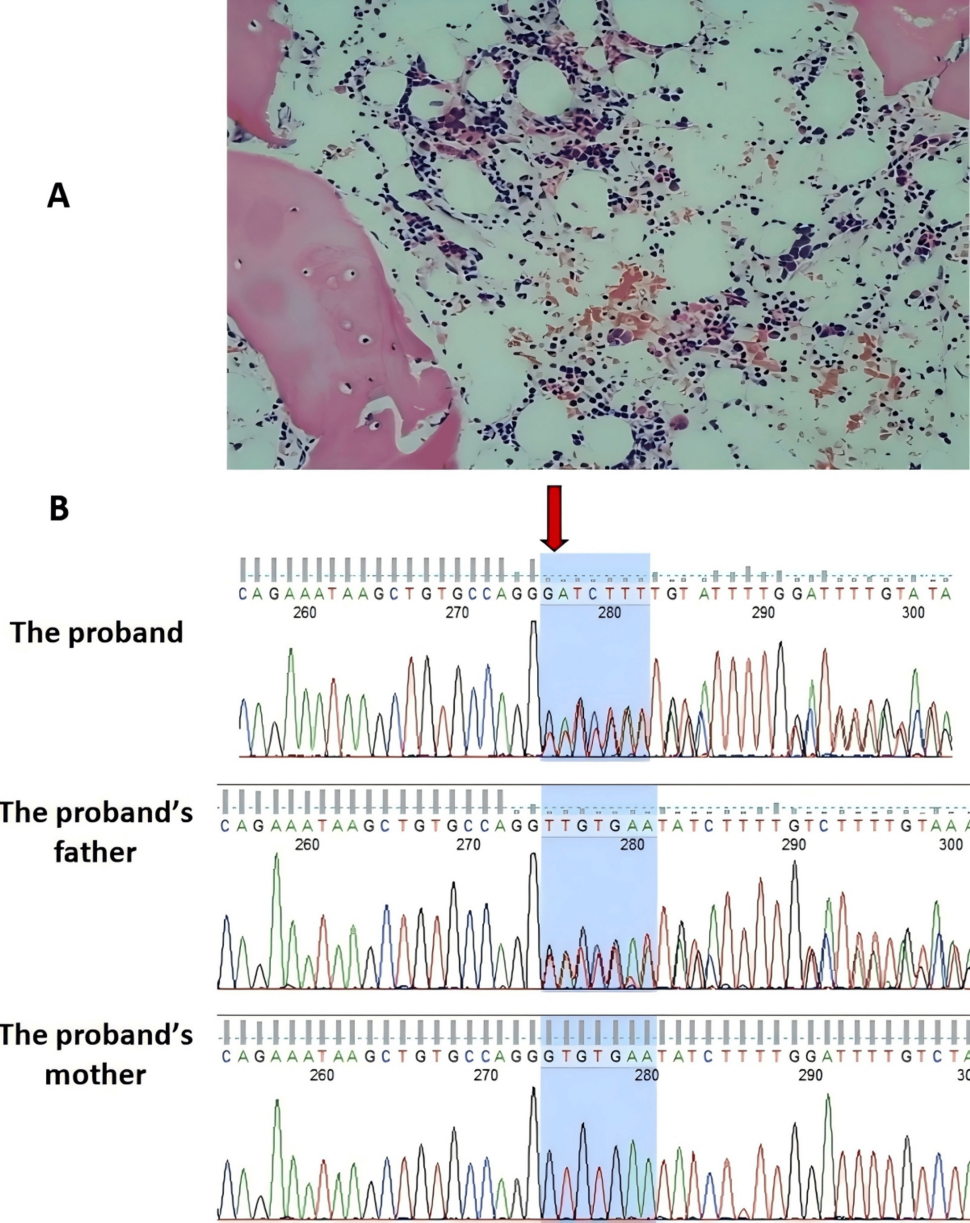
(A) Bone marrow histopathology (Hematoxylin and Eosin staining, HE ×400); (B) Representative chromatograms from Sanger sequencing of family members The images show the proband, their father, and mother in sequence. The proband and his father carried the same variant of SRP72 c.1442_1448del.

Our patient was a 14-year-old male, the only child of non-consanguineous parents. He presented to the dermatology department of Nanxishan Hospital in the Guangxi Zhuang Autonomous Region with pruritus. Blood routine examination revealed pancytopenia (hemoglobin (Hb) 7.8 g/dL, white blood cell (WBC) count 1.26 × 10^9/L, absolute neutrophil count (ANC) 0.46 × 10^9/L, platelets (PLT) 45 × 10^9/L). The reticulocyte percentage was 1.42%, and the absolute reticulocyte count was 28.30 × 10^9/L, both within the normal range. Bone marrow cytology and immunohistochemistry showed decreased bone marrow proliferation, low granulocyte proportion, increased erythrocyte proportion, and rare megakaryocytes (Figure 1A). Cytogenetics were normal.

The patient’s parents provided informed consent for genetic testing. Peripheral blood samples from the patient and his parents were collected for whole-exome sequencing (WES). DNA was extracted from white blood cells using the QIAamp DNA Blood Mini Kit (Qiagen, Hilden, Germany). WES was performed on the NovaSeq 6000 platform (Illumina, San Diego, California, US). Variants were annotated and screened using population frequency databases (dbSNP, 1000Genomes, GnomAD, ExAC, ESP) and genetic disease databases (OMIM, HGMD, ClinVar). Variants were classified according to the American College of Medical Genetics (ACMG) guidelines. Sanger sequencing was used to confirm candidate variants and inheritance patterns. Genetic testing identified a heterozygous SRP72 gene mutation (c.1442_1448del, p.Ile481Thrfs*12), a novel frameshift mutation inherited from his asymptomatic father (Figure 1B). This variant was not found in the gnomAD(v4.1.0) database or other literature, indicating it was a novel mutation. Sanger sequencing confirmed paternal inheritance. According to the ACMG guidelines, this mutation was classified as likely pathogenic (LP) (PVS1+PM2_supporting).

One month after discharge, the patient was readmitted due to fever for one day. After admission, the patient received anti-infection treatment, blood cell increase, cyclosporine capsules in combination with stanozolol tablets to promote hematopoiesis, supplementation of hematopoietic substrates (caffeic acid, vitamin B6, and folic acid), and symptomatic treatment. The patient improved and was discharged. During follow-up, the patient reported fatigue but no other abnormalities and was able to participate in learning activities normally. Some patients with *SRP72* gene mutations have hearing loss, but our patient had normal results in the hearing test.

**Figure 2:**
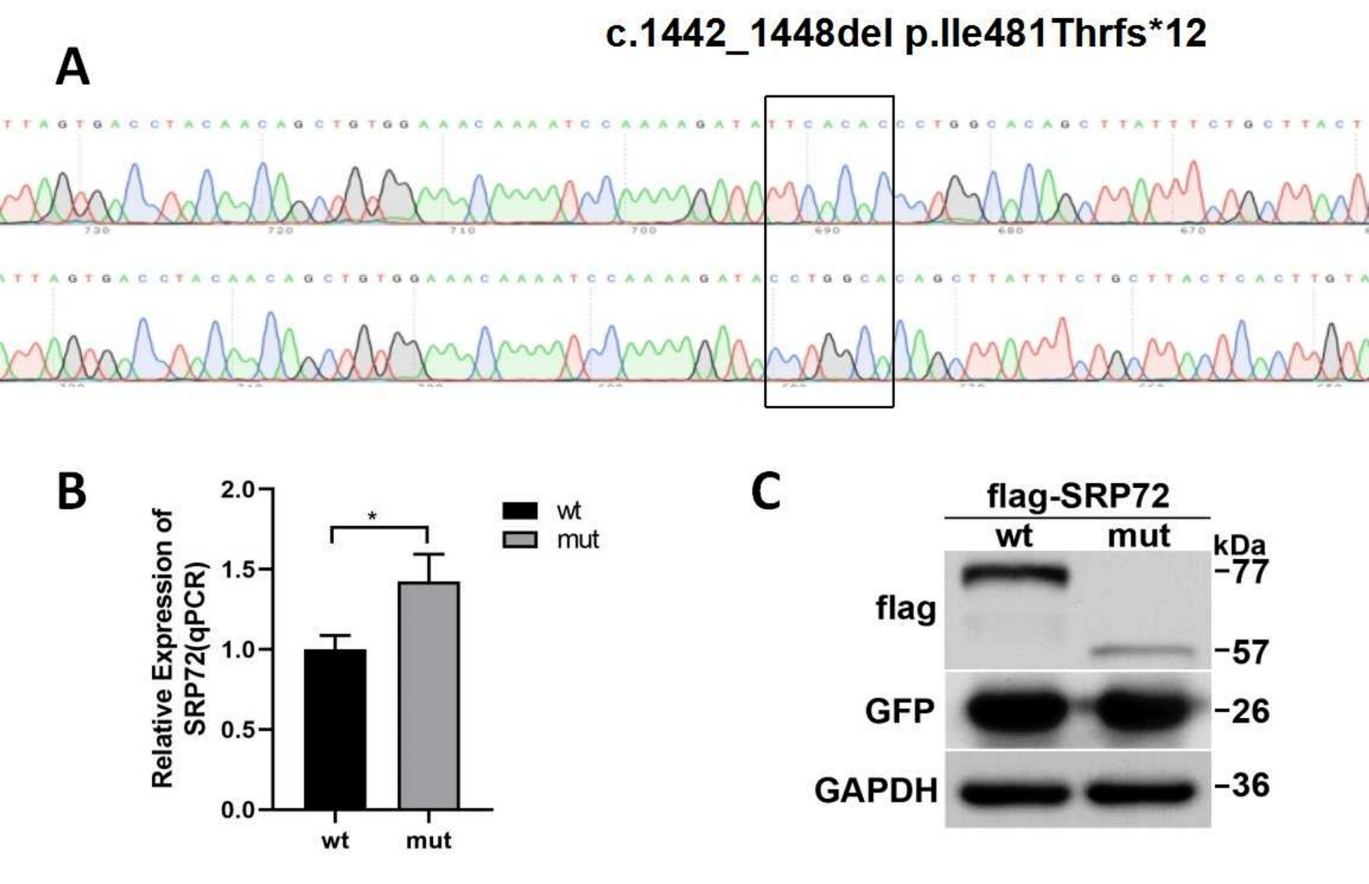
(A) SRP72-wt/mut vector construction and sequencing results; (B) Relative mRNA expression of SRP72 mutant and wild type (qPCR), the mRNA level of mutant mut was significantly upregulated (mRNA expression=1.42491,*p < 0.05); (C) Relative expression of SRP72 mutant and wild-type protein (WB), the protein level was significantly reduced (ptotein level=21% of WT,***p<0.001).

To assess the functional impact of the mutation, SRP72 was subcloned into a pEGFP-C1 expression plasmid. Plasmids expressing wild-type or c.1442_1448del (p.Ile481Thrfs*12) mutant SRP72 were designated pCMV-3XFlag-Neo(EGFP)-wt and pCMV-3XFlag-Neo(EGFP)-mut, respectively. Sanger sequencing verified sequence accuracy (Figure 2A). The primer sequences are shown in Table 1. HEK293T cells were cultured at 37 °C with 5% CO_2_ in Dulbecco’s Modified Eagle Medium (DMEM) supplemented with 10% fetal bovine serum (FBS). Cells were seeded at 5 × 10^5 per well in six-well plates and transfected with Lipofectamine™ 3000 reagent. After 48 hours, cells were collected for further analysis. RNA was extracted using Trizol (Takara Bio, Shiga, Japan) and reverse-transcribed using the PrimeScript RT Reagent Kit. Quantitative PCR (qPCR) was performed using ABI7500 (Thermo Fisher, Waltham, MA, US) with primers SRP72-qPCR-F and SRP72-qPCR-R to detect wild-type and mutant transcript levels. Protein extraction involved lysing cells and measuring protein concentration using a BSA assay kit. SDS-PAGE and Western Blotting were used to detect wild-type and mutant protein levels. Primary antibodies included FLAG-tag (Mabnus, GS20002), GFP (DIAAN, 2057), and GAPDH (CST, 2118S). In vitro expression of SRP72-MUT in the pCMV-3XFlag-Neo(EGFP) vector showed significantly upregulated mutant mRNA levels (Figure 2B) but reduced protein levels (Figure 2C). This suggests that the truncated protein is unstable, leading to decreased protein levels. We speculate that the mechanism of this mutation is loss of function.

**Table 1:** Primer sequences of wild and mutant expression plasmids and qPCR qPCR: quantitative PCR

Name	Primer Name	Primer Sequences	Length
Generation of plasmids	Neo(EGFP)-SRP72-EcoRI-F	gccgcgaattcaATGGCGAGCGGCGGCAGC	2038bp
	Neo(EGFP)-SRP72-BamHI-R	ATCgggatccTCACCAGCCACCTTTTCCAC	
	SRP72-mut-F	AAAATCCAAAAGATACCTGGCACAGCTTAT	2031bp
	SRP72-mut-R	ATAAGCTGTGCCAGGTATCTTTTGGATTTT	
qPCR	SRP72-QPCR-F	TTGGGAAAAAGTGGTTCCAG	182bp
	SRP72-QPCR-R	GGTCTTCCTCAGTCCCATCA	

## Discussion

The *SRP72* gene (*602122) has been identified in the OMIM database as a causative gene for inherited bone marrow failure syndrome 1 (IBMFS1, #614675), an autosomal dominant disorder characterized by early-onset aplastic anemia (AA) or pancytopenia in some patients, and adult-onset myelodysplasia in others. Additional clinical features, such as deafness or labyrinthitis, have also been reported in affected individuals. In the literature, the first family with *SRP72*-related IBMFS1 included four affected patients harboring the mutation c.1064_1065del (p.Thr355Lysfs19), which was considered the primary genetic cause. A second family was identified with a different mutation (c.620G>A (p.Arg207His)), presenting with myelodysplasia but without deafness. Our patient’s mutation (c.1442_1448del, p.Ile481Thrfs*12) is located in the same functional domain as the mutation in the first family, implicating the binding of *SRP72* to the *7SL RNA* [8]. This suggests that mutations in this domain may disrupt the normal function of the SRP complex, leading to hematologic abnormalities.

To further verify the relationship between *SRP72* mutations and IBMFS, we recommended genetic testing for the patient’s grandparents. However, this was declined by the patient’s parents, limiting our ability to expand the study to a larger family and assess potential risks. Despite this, only the 14-year-old patient in the family developed pancytopenia, while his asymptomatic father, who carries the same mutation, remains healthy. This raises the possibility that additional environmental factors, such as radioresistance or exposure to cytotoxic agents, may be required to trigger the development of IBMFS in carriers of *SRP72* mutations [9,10]. Our patient’s father, who is currently 46 years old and the oldest affected individual in the first family (76 years old), highlights the need for continued monitoring of carriers for potential late-onset manifestations.

BMFS can be broadly categorized into inherited (IBMFS) and acquired (ABMFS) forms, each with distinct etiologies, pathophysiological mechanisms, and clinical implications. IBMFS are caused by germline mutations that disrupt critical biological processes such as DNA repair, telomere maintenance, and ribosome biogenesis, often presenting with a constellation of hematological and non-hematological features. In contrast, ABMFS are primarily driven by external factors, such as infections, toxins, and autoimmune mechanisms, typically presenting with isolated cytopenias and lacking the characteristic congenital anomalies seen in IBMFS [11]. Both IBMFS and ABMFS pose significant clinical challenges. The genetic heterogeneity of IBMFS complicates diagnosis and management, necessitating the development of comprehensive genetic panels and improved analytical tools. For ABMFS, identifying the precise etiology and optimizing immune modulation remain key areas of research. Advances in genomics and immunotherapy hold promise for improving outcomes in both groups of disorders. Understanding these differences is crucial for the accurate diagnosis and effective management of these complex disorders.

## Conclusions

Our patient represents the third reported case, to the best of our knowledge, of pancytopenia associated with an S*RP72 *mutation but without accompanying deafness or a family history of disease. The affected individuals in previously reported studies have not required specific treatment, and our patient received symptomatic management and was discharged from the hospital. We speculate that the mutation in this gene may result in a relatively mild clinical manifestation of hematologic malignancy. *SRP72* gene mutations are extremely rare in the context of BMFS, and this case provides a valuable reference for clinicians to be more vigilant about this specific etiology. Our work expands the mutation spectrum of the *SRP72 *gene and provides valuable information for future diagnosis of IBMFS1.

## References

[1] Niewisch MR, Savage SA (2019). An update on the biology and management of dyskeratosis congenita and related telomere biology disorders. Expert Rev Hematol.

[2] Skibenes ST, Clausen I, Raaschou-Jensen K (2021). Next-generation sequencing in hypoplastic bone marrow failure: what difference does it make?. Eur J Haematol.

[3] Bluteau O, Sebert M, Leblanc T (2018). A landscape of germ line mutations in a cohort of inherited bone marrow failure patients. Blood.

[4] Wang P, Jiang W, Lai T, Liu Q, Shen Y, Ye B, Wu D (2024). Germline variants in acquired aplastic anemia: current knowledge and future perspectives. Haematologica.

[5] Elghetany MT, Punia JN, Marcogliese AN (2021). Inherited bone marrow failure syndromes: biology and diagnostic clues. Clin Lab Med.

[6] Yin J, Iakhiaeva E, Menichelli E, Zwieb C (2007). Identification of the RNA binding regions of SRP68/72 and SRP72 by systematic mutagenesis of human SRP RNA. RNA Biol.

[7] Becker MM, Lapouge K, Segnitz B, Wild K, Sinning I (2017). Structures of human SRP72 complexes provide insights into SRP RNA remodeling and ribosome interaction. Nucleic Acids Res.

[8] Kirwan M, Walne AJ, Plagnol V (2012). Exome sequencing identifies autosomal-dominant SRP72 mutations associated with familial aplasia and myelodysplasia. Am J Hum Genet.

[9] Prevo R, Tiwana GS, Maughan TS, Buffa FM, McKenna WG, Higgins GS (2017). Depletion of signal recognition particle 72kDa increases radiosensitivity. Cancer Biol Ther.

[10] D'Altri T, Schuster MB, Wenzel A, Porse BT (2019). Heterozygous loss of Srp72 in mice is not associated with major hematological phenotypes. Eur J Haematol.

[11] Leguit RJ, van den Tweel JG (2010). The pathology of bone marrow failure. Histopathology.

